# 3D-Printed Biomaterial Testing in Response to Cryoablation: Implications for Surgical Ventricular Tachycardia Ablation

**DOI:** 10.3390/jcm12031036

**Published:** 2023-01-29

**Authors:** Mara Candelari, Ida Anna Cappello, Luigi Pannone, Cinzia Monaco, Edoardo Bori, Giacomo Talevi, Robbert Ramak, Mark La Meir, Ali Gharaviri, Gian Battista Chierchia, Carlo de Asmundis, Bernardo Innocenti

**Affiliations:** 1 Heart Rhythm Management Centre, Postgraduate Program in Cardiac Electrophysiology and Pacing, Universitair Ziekenhuis Brussel-Vrije Universiteit Brussel, European Reference Networks Guard-Heart, 1090 Brussels, Belgium; 2 BEAMS Department (Bio Electro and Mechanical Systems), Université Libre de Bruxelles, 1050 Brussels, Belgium; 3 Cardiac Surgery Department, Universitair Ziekenhuis Brussel-Vrije Universiteit Brussel, 1090 Brussels, Belgium

**Keywords:** arrhythmia treatment, biomaterial tests, cryoablation, 3D surgical guide

## Abstract

**Background:** The lack of thermally and mechanically performant biomaterials represents the major limit for 3D-printed surgical guides, aimed at facilitating complex surgery and ablations. **Methods:** Cryosurgery is a treatment for cardiac arrhythmias. It consists of obtaining cryolesions, by freezing the target tissue, resulting in selective and irreversible damage. MED625FLX and TPU95A are two biocompatible materials for surgical guides; however, there are no data on their response to cryoenergy delivery. The study purpose is to evaluate the biomaterials’ thermal properties, examining the temperature changes on the porcine muscle samples (PMS) when the biomaterials are in place during the cryoablation. Two biomaterials were selected, MED625FLX and TPU95A, with two thicknesses (1.0 and 2.5 mm). To analyze the biomaterials’ behavior, the PMS temperatures were measured during cryoablation, firstly without biomaterials (control) and after with the biomaterials in place. To verify the biomaterials’ suitability, the temperatures under the biomaterial samples should not exceed a limit of −30.0 °C. Furthermore, the biomaterials’ geometry after cryoablation was evaluated using the grid paper test. **Results:** TPU95A (1.0 and 2.5 mm) successfully passed all tests, making this material suitable for cryoablation treatment. MED625FLX of 1.0 mm did not retain its shape, losing its function according to the grid paper test. Further, MED625FLX of 2.5 mm is also suitable for use with a cryoenergy source. **Conclusions:** TPU95A (1.0 and 2.5 mm) and MED625FLX of 2.5 mm could be used in the design of surgical guides for cryoablation treatment, because of their mechanical, geometrical, and thermal properties. The positive results from the thermal tests on these materials and their thickness prompt further clinical investigation.

## 1. Introduction

Since 3D printing has the potential to provide designs that are patient specific, with highly accurate manufacturing, and rapid production, 3D printing is a suitable technology to build surgical guides to facilitate complex surgery in several medical fields.

However, among the major limitations for biomedical manufacturing is the lack of biomaterials, or polymers and hydrogels suitable for 3D printing, that are both biocompatible and performing from a biomechanical and thermal standpoint [1]. Another limitation is the lack of data on the response of such materials to cryoenergy, that is often the preferred choice for cardiac ablation.

Among the different surgical and therapeutic procedures performed in the cardiac field, cryosurgery is considered among the optimal treatments for cardiac arrhythmias, by obtaining contiguous, uniformly transmural cryolesions, resulting in the irreversible damage of the arrhythmogenic tissue. Indeed, cryoablation lesions are the consequence of freezing and thawing the target tissue, while laser, radiofrequency, ultrasound and microwave energies create lesions by a temperature increase in the target tissue [2].

The effectiveness of cryolesions relies on cryocatheter temperature and dimension, target myocardium baseline temperature and time (duration) of cryothermal freeze (application); myocardial cell damage occurs during the cryoablation cycle consisting of the freezing and thawing phases [3,4,5]. The critical temperature for irreversible damage in myocardium is −30.0 °C [6,7,8]. During the freezing phase, a low temperature determines ice crystal creation, which destroys the cell membrane. Furthermore, the thawing phase leads to microvascular stasis around the cryolesions, causing cell death. The depth of the ultimate cryolesion depends on the following: the time of the frozen tissue thawing, the volume of cells, and repetitions of the freeze–thaw cycle [9,10]. However, a limitation of previous studies is the lack of a tool aiming at guiding the surgeon towards the culprit patient-specific arrhythmogenic area.

Since the recent advances in the cardiac field towards personalized 3D-printing for ventricular tachycardia ablation with cryo-technique [11], MED625FLX and TPU95A are two promising biocompatible materials for surgical guides, aimed at cryoablation treatment [12]. However, because there are no data on their response to cryoenergy delivery during epicardial cryo-treatment using these materials, this study aims at evaluating thermal properties and mechanical and geometrical alterations of MED625FLX and TPU95A during cryoablation simulation. Thermal evaluation of biocompatible materials has been performed only in response to radiofrequency [13] and there are no data on the effect of cryoenergy. However, thermal energy might be associated with damage of epicardial structures such as coronary arteries. This is of utmost clinical importance as epicardial ablation is often used with a cryocatheter for its safety profile. Thus, evaluating the effect of cryoenergy is necessary before any clinical experience with 3D-printed surgical guides.

## 2. Material and Methods

### 2.1. Procedure

The cryocatheter is composed of a cooling electrode tip, connected to a gas tube (usually the nitrous oxide gas). Ablation is the result of pressurized cryorefrigerant delivery to the catheter tip via an injection tube; this allows to maximize the temperature drop through the Joule–Thompson effect, and to accelerate the phase change of liquid to-gas for a rapid tip cooling [14]. The 4 mm-diameter-tip probe is composed of a shaft, a handle, a thermocouple, an inlet tube, and is designed to allow access to varying anatomy and anatomical sites.

In this study, the AtriCure CryoICE System (Coolrail, Atricure Inc., Mason, OH, USA) was selected as the ablation catheter (cryoablation) to investigate two 3D bio-printable materials thermal properties: MED625FLX (Stratasys Ltd., Edina, MN, USA) and TPU95A (Ultimaker B.V., Utrecht, The Netherlands). It was set at −70.0 °C for a cryoablation session of 30.0 s as per previous clinical evaluation and instruction for use (IFU) [15].

Briefly, MED625FLX is a biocompatible material, with characteristics of flexibility, lightweight, and already certified for contact with body, in orthodontic. This material is approved for dental use and skin contact. However, there are no data about cardiac use during ablation. MED625FLX was tested according to the International Organization for Standardization (ISO) requirements for biological tests [16].

The thermoplastic polyurethane material (TPU) is characterized by a broad range of mechanical properties, excellent physical properties, and biocompatibility, which make the TPU suitable for this study [17]. Moreover, the TPU is used in biomedical field for different applications, such as: blood bags, vascular catheters, and implants targeting both soft and hard tissues [18]. For this study, TPU95A was selected due to its mechanical properties which were previously evaluated by Ultimaker et al. [19].

MED625FLX was printed using the Polyjet technique by Objet260 Connex1 3D printer (Stratasys Ltd., Edina, MN, USA), while TPU95A was printed through Fused Deposition Modelling (FDM) by the Anycubic Mega Zero 2.0 3D printer (Shenzhen Anycubic Technology Co. Ltd., Shenzhen, China).

### 2.2. Experimental Test

**Figure 1:**
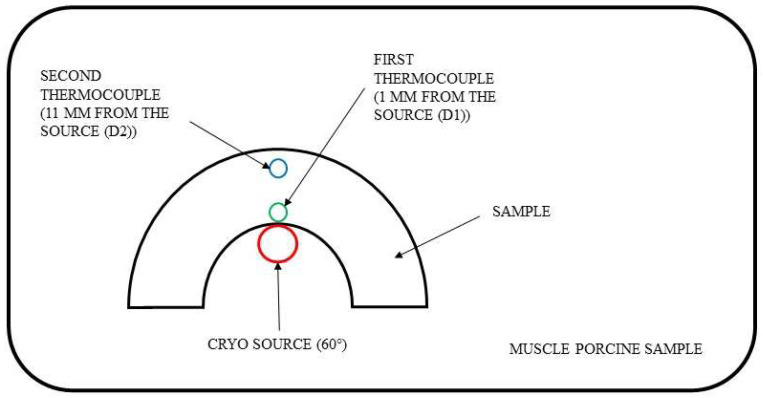
Schematic diagram of experimental tests. The red circle represents the point of cryo-application (with a 60° cryoprobe–tissue angle); instead, the green circle represents the distance of 1.0 mm from the cryo-application (D_1_), where the first thermocouple for temperature measurement is located and the blue circle represents a distance of 11.0 mm from cryo-application (D_2_), where the second thermocouple for temperature measurement is located.

The experimental test workflow is already explained in detail in our previous study on radiofrequency ablation simulation [13], Figure 1. The previous study discusses mechanical and geometrical changes in MED625FLX and TPU95A in response to radiofrequency ablation. Indeed, the goal of this study is to evaluate the changes in these materials in response to a cryoablation energy source. In order to verify if MED625FLX and TPU95A act as conductors or as insulants, depending on the layer, semicircular-shaped samples with thicknesses of 1.0 and 2.5 mm for each material were built to perform the experimental tests. The thicknesses were chosen for a proper tradeoff between flexibility and resistance; indeed, a thicker layer would make the material stiffer, while a too thin layer could lead to break. The semicircular material samples were modelled with an external diameter of 45.0 mm and an internal diameter of 15.0 mm, for a total of 4 samples (MED625FLX of 1.0 and 2.5 mm; TPU95A of 1.0 and 2.5 mm). The schematic diagram of the experimental tests is summarized in Figure 1, representing how the samples, the source and the two thermocouples are placed on the muscle porcine sample.

Since they were for surgical use, to avoid infections, after 3D printing and cleaning, MED625FLX and TPU95A must be sterilized. Therefore, according to the sterilization methods available in the hospital, the material samples underwent the Vaporized Hydrogen Peroxide (VHP) Gas Sterilization. VHP consists of first cleaning at +93.0 °C for 1 h and 30 min, and then filling the sterilization machine with H_2_O_2_ (Sterrad Sterilization System of Johnson & Johnson) at +45.0 °C for 40 min, where the gas penetrates the surface [20].

Nevertheless, even though surgical sterility for the experimental tests was not needed, we decided to analyze the sterilized materials, because the thermal and mechanical features could be altered by the high temperatures of VHP process.

**Figure 2:**
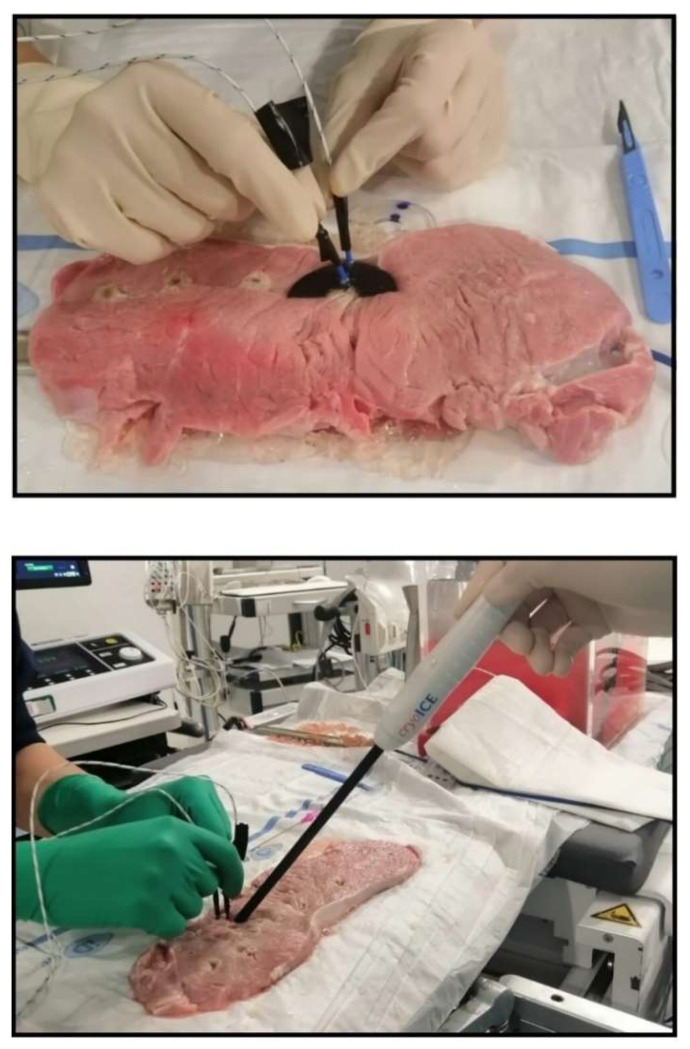
Experimental tests. Thermal test: upper panel. Biomaterial and thermocouple placement: lower panel. cryoICE catheter in place.

To perform the cryothermal tests, porcine muscle samples (PMS) with a 10.0 mm thickness were analyzed—the surgical guide is built for ventricles and the PMS thickness is similar to human ventricular wall (Figure 2) [21]. Moreover, to mimic the physiological human conditions, PMS were immersed in a Plexiglass box with +37.0 °C water filling (body temperature). On top of all experimental tests, both materials were assumed to be biocompatible, respecting the requirement for biomaterials [22,23], as shown in Table 1. The thermometer used was the VOLTCRAFT PL-125-T4 with a sensor type K, a tip of 4.0 mm, a temperature range between −200.0 and +1372.0 °C and an accuracy of ±1.0 °C. Figure 2 (upper panel) shows how the thermocouples were positioned inside the sample holes D1 and D2. Figure 2 (lower panel) shows an example of a unipolar RFA on a TPU95A sample of 2.5 mm.

The thermal test consists of two phases: (1) evaluation of freezing propagation, measuring the temperatures of PMS, through two thermocouple probes, placed at 1.0 mm (D_1_) and 11.0 mm (D_2_) of distance from the cryoablation tip; and (2) analysis of eventual mechanical and geometrical alterations after cryoablation procedure, and if the biomaterials act as thermal insulant or conductor. Indeed, if the material becomes cooler, acting as a thermal conductor, and reaches −30.0 °C, this leads to unintended cell damage; however, if the material behavior is typical of a thermal insulant, this may protect the underlying tissue from low temperatures. To ensure safe conditions, avoiding any tissue damage, the temperature under the material sample must be above −30.0 °C during cryoablation. This is necessary during cardiac cryoablation treatment for a 3D surgical guide application.

For a comparative analysis of the results, the control temperatures were firstly recorded without material sample placement on the PMS (control). All samples underwent four consecutive applications of 30 s to analyze the role of repetitive cryoablation applications. The temperature measurements were recorded at D_1_ and D_2_ of distance from the cryo-source. The temperature was measured accordingly during continuous cryoablation, and four time references were considered: 0, 10, 15 and 30 s. During the tests, the samples of both materials were located on PMS, and underwent 4 ablation sessions of 30 s; the temperature values were measured in D_1_ and D_2_ at 0, 10, 15 and 30 s. Each temperature assessment was repeated three times.

Each cryoablation application was delivered in a different place on the PMS to avoid the new measurement being affected by the previous cryoablation. Clinical testing was repeated for both TPU95A and MED625FLX of 1.0 mm and 2.5 mm. At the end of each session of 4 cryoapplications, the biomaterial samples of TPU95A and MED625FLX of both thicknesses were positioned on a grid paper to evaluate any eventual shape changes after cryoablation.

Low-temperature differential scanning calorimetry (DSC) was used to analyze both MED625FLX, using a sample of 8.7200 mg, and TPU95A, using a sample of 8.4700 mg, after VHP sterilization. Accuracy was evaluated with repeatability of temperature measurements and error bars for each thermocouple.

For the assessment workflow, a specific number was assigned to each biomaterial sample: sample “1” to MED625FLX of 2.5 mm, sample “2” to TPU95A of 2.5 mm, sample “3” to MED625FLX of 1.0 mm and sample “4” to TPU95A of 1.0 mm. To verify the biomaterials’ thermal suitability: (1) the temperature under the biomaterial samples should not exceed −30.0 °C; (2) the temperature difference between material samples and controls should not be statistically significant; and (3) the cryoablation should not alter the biomaterials’ geometry.

### 2.3. Statistical Analysis

To compare the biomaterials’ response to 30 s-cryoablation sessions with respect to the control, the statistical analysis was performed measuring the temperature values at 0, 10, 15, and 30 s of underlying PMS, by distinguishing different material thicknesses (1.0 and 2.5 mm) and different distances (D_1_ and D_2_). The Shapiro–Wilk test was used for all variables to test for normality. The mean ± standard deviation were used for normally distributed variables and they were compared through an unpaired t-test, while the median (interquartile range) was used for non-normally distributed variables and they were compared by the Mann–Whitney test or the Wilcoxon signed-rank test. Frequencies and percentages were used for categorical variables and compared by Chi-squared test or Fisher’s exact test. A *p*-value less than 0.05 was considered statistically significant. The analysis was performed using R software version 3.6.2 (R Foundation for Statistical Computing, Vienna, Austria).

## 3. Results

The results of the thermal tests illustrate the freezing propagation on the PMS in response to cryoablation. The control temperature measurements are summarized in Table 2, while Table 3 and Table 4 report the evaluation of temperatures with the material samples in place (TPU95A and MED625FLX) during cryoablation, respectively. All temperatures were measured at 0, 10, 15 and 30 s for both distances, D_1_ and D_2_.

**Figure 3:**
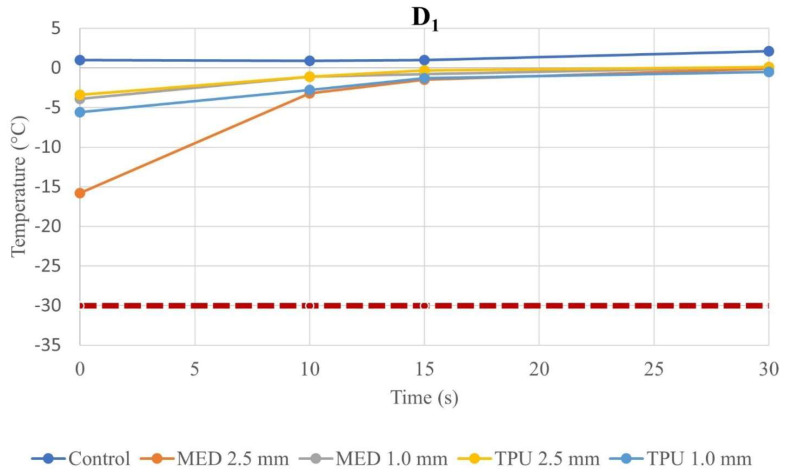
Temperature–time curve of control and biomaterial samples in D_1_. The graph represents the temperature–time curve at D_1_ from the cryoablation catheter of the control, MED625FLX and TPU95A of 1.0 and 2.5 mm of thickness; the dashed line indicates the threshold set at −30.0 °C. Below the threshold, the freezing causes cell death.

**Figure 4:**
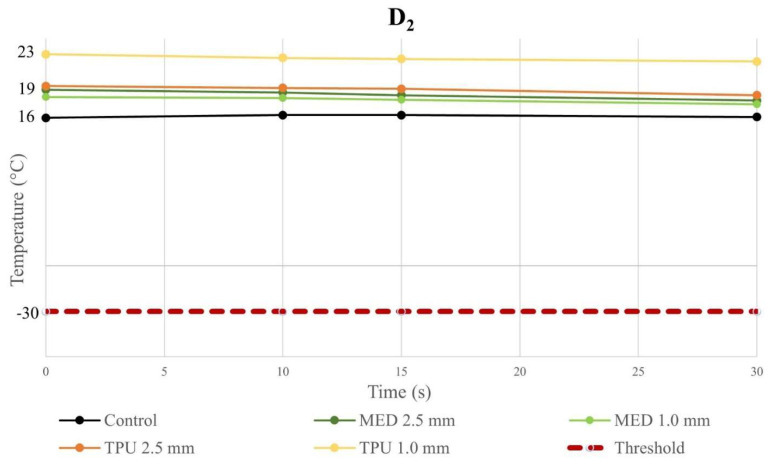
Temperature–time curve of control and biomaterial samples in D_2_. The graph represents the temperature–time curve at D_2_ from the cryoablation catheter of the control, MED625FLX and TPU95A of 1.0 and 2.5 mm of thickness; the dashed line indicates the threshold set at −30.0 °C. Below the threshold, freezing causes cell death.

**Figure 5:**
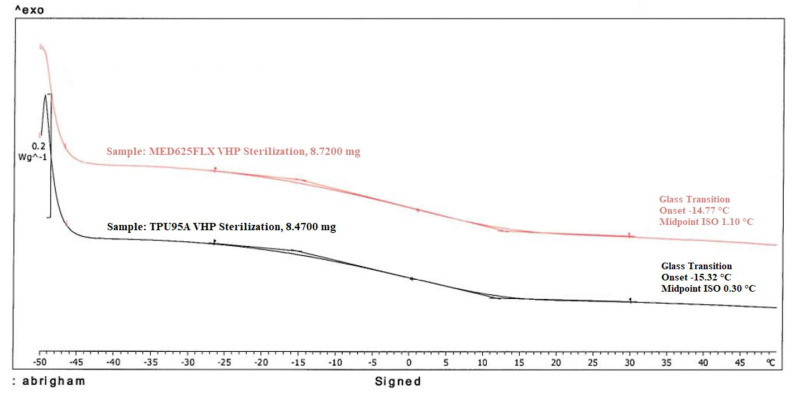
Low-temperature DSC of biomaterials. The graph represents the low-temperature DSC behavior of both MED625FLX (with a sample of 8.7200 mg) and TPU95A (with a sample of 8.4700 mg) after VHP sterilization.

At each time instant, the temperature with the biomaterial samples in position was not different compared with the control, for D_1_ and D_2_, independently from the thickness (Tables S1 and S2). Because of the stress induced on the material by cryoablation, the temperatures at the fourth (last) application were of particular interest and the values were reported in the line charts; in particular, these charts illustrate the evolution of temperatures during time of the fourth cryo-application for both the control and material samples. The temperature trend at D_1_ is represented in Figure 3 and, the temperature behavior at D_2_ is represented in Figure 4; in both graphs, the dashed lines define the threshold, set at −30.0 °C, as previously defined. All the measured temperatures with the different tested materials and thicknesses were below the safety threshold. Moreover, low-temperature DSC was used to analyze for both MED625FLX, using a sample of 8.7200 mg, and TPU95A, using a sample of 8.4700 mg, after VHP sterilization. The results are summarized in Figure 5.

**Figure 6:**
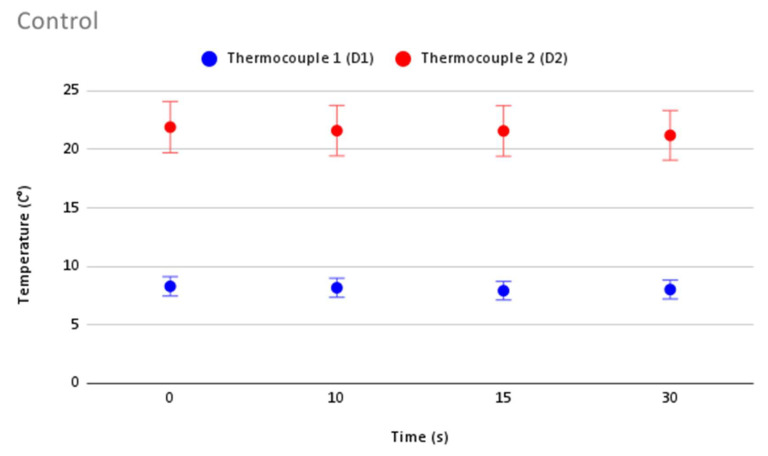
Repeatability of temperature measurements and error bars for the control. Repeatability of temperature measurements and error bars for the control.

**Figure 7:**
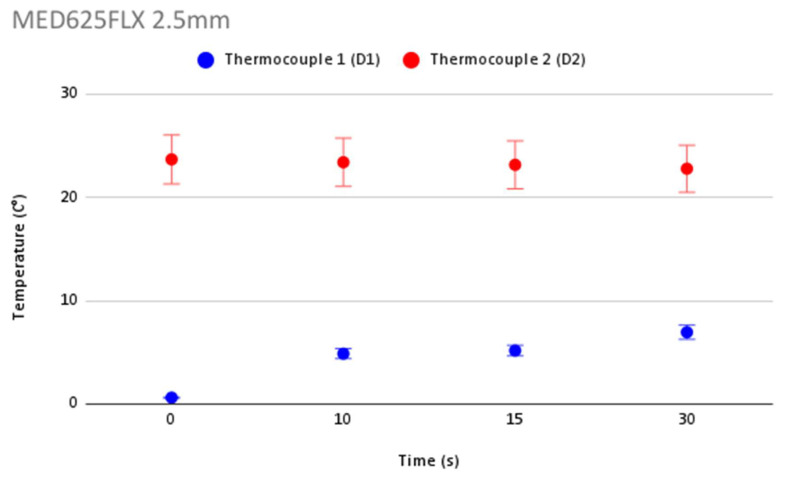
Repeatability of temperature measurements and error bars for MED625FLX of 2.5 mm. Repeatability of temperature measurements and error bars for MED625FLX of 2.5 mm.

**Figure 8:**
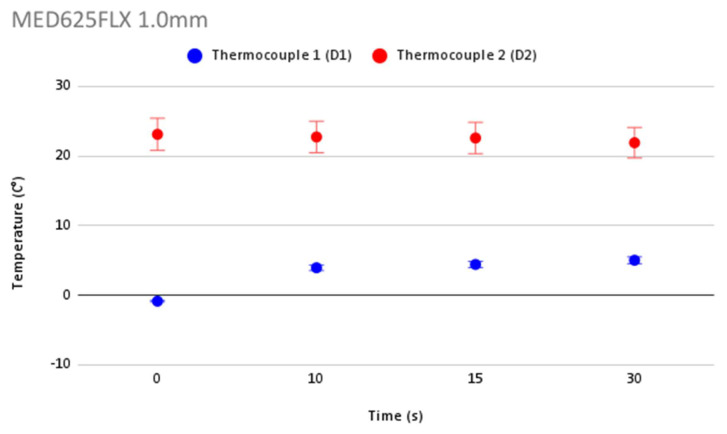
Repeatability of temperature measurements and error bars for MED625FLX of 1.0 mm. Repeatability of temperature measurements and error bars for MED625FLX of 1.0 mm.

**Figure 9:**
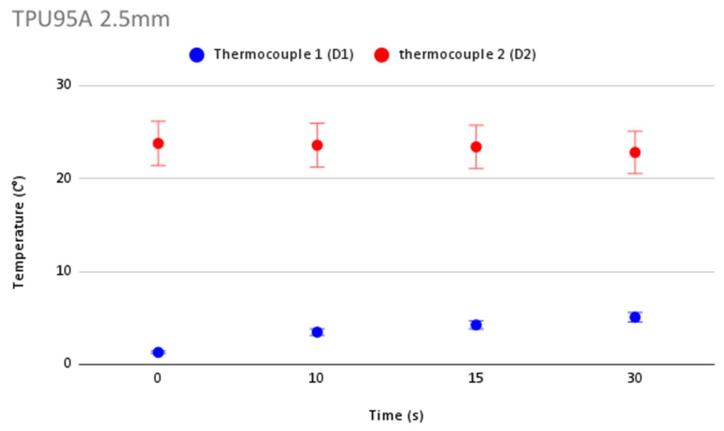
Repeatability of temperature measurements and error bars for TPU95A of 2.5 mm. Repeatability of temperature measurements and error bars for TPU95A of 2.5 mm.

**Figure 10:**
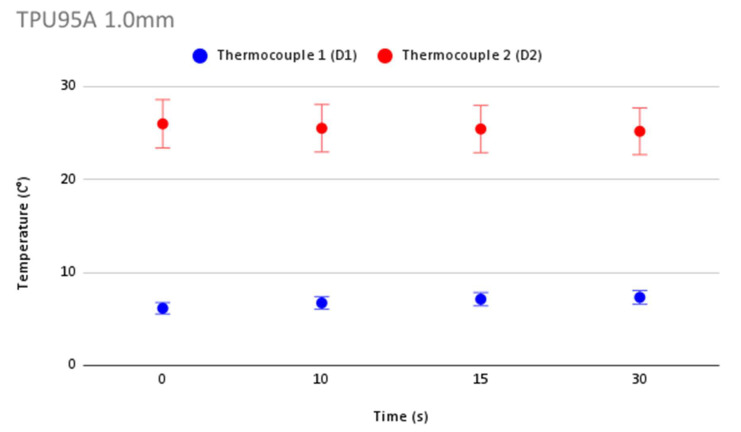
Repeatability of temperature measurements and error bars for TPU95A of 1.0 mm. Repeatability of temperature measurements and error bars for TPU95A of 1.0 mm.

In order to validate the accuracy in temperature measurement during cryoablation, Figure 6 shows repeatability of temperature measurements and the error bars for each thermocouple for the control. Figure 7, Figure 8, Figure 9 and Figure 10 show the repeatability of temperature measurements and the error bars for each thermocouple for MED625FLX of 2.5 mm, MED625FLX of 1.0 mm, TPU95A of 2.5 mm and TPU95A of 1.0 mm, respectively.

**Figure 11:**
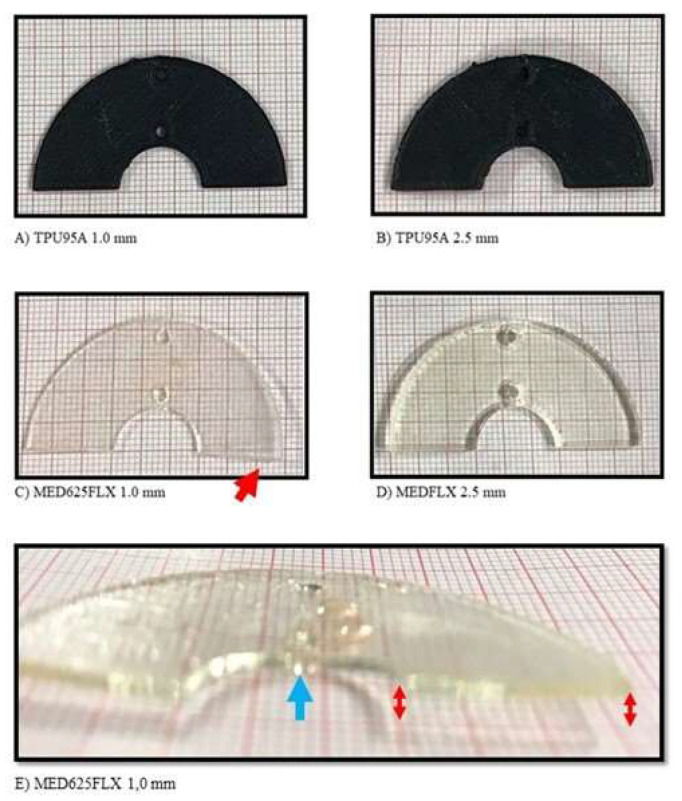
The macroscopic biomaterial changes in geometry after the cryoablation catheter. The macroscopic material changes in geometry after the cryo-application are shown in the figure; (**A**) TPU95A of 1.0 mm of thickness; (**B**) TPU95A of 2.5 mm of thickness; (**C**) MED625FLX of 1.0 mm of thickness; (**D**) MED625FLX of 2.5 mm of thickness; (**E**) lateral view of MED625FLX of 1.0 mm. The red arrows indicate the material deflection; the blue arrow indicates the break point in correspondence to the cryoablation.

Finally, the experimental results of the second phase regarding the macroscopic changes on material samples are shown in Figure 11. From the picture, representing MED625FLX and TPU95A of both thicknesses, placed on a grid paper, it is possible to appreciate the macroscopic freezing and thawing effects due to cryoablation. No visible mechanical alterations in MED625FLX of 2.5 mm, TPU95A of 2.5 mm and TPU95A of 1.0 mm were observed. While cryoablation using MED625FLX of 1.0 mm caused middle bending and led to sample break with a loss of function.

## 4. Discussion

During the last decade, 3D printing has been developed for personalized surgical guides in medical fields such as orthopedic, orthodontist, as well as for cardiac surgery. However, the major limitation remains the lack of 3D printing-suitable biomaterials, polymers and hydrogels that are both biocompatible and have good performance from biomechanical and thermal perspectives [1]. Recently, advances have been made in the cardiac field such as 3D-printed surgical guides for ablation treatment. Two promising materials have been described, namely MED625FLX and TPU95A [2].

However, the behavior of these materials in response to cryoablation has not been evaluated. This is of utmost clinical importance because, if such a material becomes cooler, reaching −30.0 °C, it can determine unintended cell damage; however, if the material behavior is similar to an insulant, it may protect the tissue from effective freezing. Aiming at targeting tissue for cryoablation, a surgical guide material should avoid freezing the underlying tissue, leading to unintentional damage.

For this reason, this work aims at evaluating the thermal properties of TPU95A and MED625FLX, in response to a clinically approved cryoablation catheter, analyzing geometrical and mechanical changes in the materials and measuring the propagation of temperature in the underlying tissue.

The hereby presented experimental tests have been conducted analyzing the temperature of PMS under four semicircular-shaped samples, printed in both materials. The PMS were characterized by 10.0 mm of thickness, mimicking the cardiac tissue; indeed, given that the surgical guide is designed for ventricles, the PMS thickness is similar to the wall of a standard human ventricle. Moreover, two different thicknesses (1.0 and 2.5 mm) were analyzed, because thickness and material morphology influence mechanical properties [13].

In detail, every trial session with MED625FLX and TPU95A biomaterial samples has demonstrated no statistical difference in temperature, in D_1_ and D_2_, with respect to the control. Nevertheless, each session remained above the threshold (−30.0 °C), guarantying the safe conditions. By comparing D_1_ and D_2_, independently of the thickness, the measured temperature propagation in the underlying tissue, for all material samples, are directly proportional to the distance from the source. Therefore, when the biomaterials were in place, the temperature propagation maintained the safe parameters, remaining far above the threshold (min value −20.8 °C).

Analyzing the temperatures values, the last application was of utmost importance, secondary to the stress of a material to cryoablation, thus, the changes of temperature at fourth application were evaluated. By these findings, it was possible to verify that the temperature measurements in D_1_ and D_2_, regardless of material thickness, remained above the defined threshold (−30.0 °C), ensuring safe conditions.

In the second phase of this study, concerning the analysis of eventual mechanical and geometrical alterations after cryo-application, both 1.0 and 2.5 mm of TPU95A samples were not affected by any macroscopically geometrical changes; however, the MED625FLX showed a different response to cryoablation depending on the thicknesses. Indeed, the 2.5 mm sample showed no material changes in geometry, while the cryoablation changed the 1.0 mm sample shape, causing middle bending and leading to sample break with a loss of function.

Since TPU95A passed all experimental tests, it can be considered a suitable material for cryoablation treatment. MED625FLX of 2.5 mm is also suitable for use with a cryoenergy source. However, MED625FLX of 1.0 mm did not retain its shape, losing its function according to the grid paper test.

## 5. Limitations

The main limitation of this study is in its in vitro design. It is necessary for preliminary testing; however, in vivo studies on animal models and the beating heart are awaited to confirm the mechanical and thermal properties of 3D surgical guides. The combined use of cryoenergy and radiofrequency at the same time has not been tested. However, this occurrence is unlikely from a clinical standpoint. Geometrical macroscopic changes after applications were discussed in this paper. Microstructure changes were outside the scope of this work and warrant further evaluation.

## 6. Conclusions

TPU95A (1.0 and 2.5 mm) and MED625FLX of 2.5 mm are suitable for future prototype modelling of cardiac surgical guides, due to their mechanical, geometrical, and thermal properties. All materials showed a good safety profile with temperatures never exceeding the safety threshold. Concerning clinical use, further clinical investigations are eagerly awaited. In particular, studies on animal models and the beating heart are eagerly awaited to confirm these results in in vivo settings. Furthermore, research will be directed towards testing the material release on the underlying tissue.

## Figures and Tables

**Table 1:** Biomaterials requirements.

Test	Standard
Cytotoxicity	EN ISO 10993-5:2009
Irritation	EN ISO 10993-10:2013
Delayed-type hypersensitivity	EN ISO 10993-10:2013
Material mediated pyrogenicity (FDM USP <151>)	EN ISO 10993-11:2009
Acute systemic toxicity	EN ISO 10993-11:2009
Chemical characterization	EN ISO 10993-18:2009
Allowable limits for leachable substances	EN ISO 10993-17:2009
USP plastic class VI	USP 34 <88>
Genotoxicity	EN ISO 10993-3:2014

**Table 2:** Temperature measurement of the control during cryo-application.

Control	Temperature in D_1_ (°C)				Temperature in D_2_ (°C)			
Control *	14.5	14.2	12.9	11.2	23.0	22.4	22.6	22.4
Control **	9.7	9.1	8.7	8.0	23.9	23.5	23.4	22.7
Control ***	8.0	8.5	9.1	10.8	24.4	23.9	23.7	23.3
Control ****	1.0	0.9	1.0	2.1	16.3	16.6	16.6	16.4
Time	0 s	10 s	15 s	30 s	0 s	10 s	15 s	30 s

The control refers to the temperature measurement of the porcine muscle sample during cryo-application at D_1_ (1.0 mm) and D_2_ (11.0 mm). Each star (*) indicate the number of performed applications.

**Table 3:** Temperature measurements using MED625FLX samples of 1.0 and 2.5 mm for the cryo-ablation procedure.

MED625FLX	Sample N°	Temperature in D_1_ (°C)				Temperature in D_2_ (°C)			
2.5 mm	1 *	11.0	10.3	10.2	11.3	29.0	28.8	28.5	28.3
	1 **	16.0	15.4	14.2	17.4	27.0	26.8	26.7	26.3
	1 ***	−8.7	−3.0	−2.2	−0.8	19.3	18.9	18.6	18.3
	1 ****	−15.8	−3.2	−1.5	−0.1	19.4	19.1	18.8	18.2
1.0 mm	3 *	5.9	5.7	6.1	6.9	29.0	28.5	28.4	27.2
	3 **	15.4	15.2	15.2	15.3	27.3	27.2	27.0	26.8
	3 ***	−20.8	−4.0	−2.8	−2.2	17.5	16.7	16.6	15.8
	3 ****	−3.9	−1.1	−0.8	0.1	18.6	18.5	18.3	17.8
Time		0 s	10 s	15 s	30 s	0 s	10 s	15 s	30 s

MED625FLX samples of 1.0 and 2.5 mm are tested during the cryo-ablation procedure. The temperatures of underlying tissue were measured at different distances, D_1_ (1.0 mm) and D_2_ (11.0 mm), from the source at 0, 10, 15 and 30 s after cryo-ablation. Each star (*) identifies the number of cryo-ablations performed.

**Table 4:** Temperature measurements under TPU95A samples of 1.0 and 2.5 mm for the cryo-ablation procedure.

TPU95A	Sample N°	Temperature in D_1_ (°C)				Temperature in D_2_ (°C)			
2.5 mm	2 *	12.2	12.4	12.6	13.4	27.9	27.6	27.4	27.0
	2 **	7.5	6.8	6.8	7.8	25.2	25.2	24.9	24.1
	2 ***	−11.1	−4.2	−2.1	−1.0	22.2	21.9	21.8	21.3
	2 ****	−3.4	−1.1	−0.3	0.1	19.8	19.6	19.5	18.8
1.0 mm	4 *	11.6	10.6	9.8	9.5	28.9	28.6	28.4	27.9
	4 **	18.2	18.2	18.1	17.7	28.0	27.9	27.8	27.5
	4 ***	0.4	0.9	1.9	2.6	23.7	22.6	22.7	22.8
	4 ****	−5.6	−2.8	−1.3	−0.5	23.3	22.9	22.8	22.5
Time		0 s	10 s	15 s	30 s	0 s	10 s	15 s	30 s

TPU95A samples of 1.0 and 2.5 mm are tested during the cryo-ablation procedure. The temperatures of underlying tissue were measured at different distances, D_1_ (1.0 mm) and D_2_ (11.0 mm), from the source at 0, 10, 15 and 30 s after cryo-ablation. Each star (*) identifies the number of cryo-ablations performed.

## Data Availability

Data are contained within this article or Supplementary Materials.

## Supplementary Materials

The following supporting information can be downloaded at: https://www.mdpi.com/article/10.3390/jcm12031036/s1, Table S1: Statistical analysis of temperature means between control and PMS under the biomaterial samples in D_1_; Table S2: Statistical analysis of temperature means between control and PMS under the biomaterial samples in D_2_.
