# Supplementary material for: 3D-Printed Biomaterial Testing in Response to Cryoablation: Implications for Surgical Ventricular Tachycardia Ablation
Source: J Clin Med. 2023 Jan 29;12(3):1036. doi: 10.3390/jcm12031036 (PMC9918061; doi:10.3390/jcm12031036)
Supplement: Supplementary file 1 [file jcm-12-01036-s001.zip › jcm-2033083-supplementary.pdf]

3D-Printed Biomaterials Testing in Response to Cryoablation

SUPPLEMENTARY MATERIALS

Supplementary Table S1. Statistical analysis of temperatures means between control and PMS under the biomaterial samples in D_1

Distance	Time	Biomaterial	Control mean (SD)	Biomaterial mean (SD)	pvalue
**D_1**	**0 s**	**Med_2.5 mm**	8.3 (5.6)	0.6 (15.3)	0.382
	**10 s**	**Med_2.5 mm**	8.2 (5.5)	4.9 (9.4)	0.568
	**15 s**	**Med_2.5 mm**	7.9 (5.0)	5.2 (8.3)	0.590
	**30 s**	**Med_2.5 mm**	8.0 (4.2)	7.0 (8.9)	0.834
	**0 s**	**Med_1.0 mm**	8.3 (5.6)	-0.8 (15.5)	0.308
	**10 s**	**Med_1.0 mm**	8.2 (5.5)	3.9 (8.5)	0.437
	**15 s**	**Med_1.0 mm**	7.9 (5.0)	4.4 (8.1)	0.491
	**30 s**	**Med_1.0 mm**	8.0 (4.2)	5.0 (7.9)	0.526

	**0 s**	**Tpu_2.5 mm**	8.3 (5.6)	1.3 (10.5)	0.285
	**10 s**	**Tpu_2.5 mm**	8.2 (5.5)	3.5 (7.5)	0.352
	**15 s**	**Tpu_2.5 mm**	7.9 (5.0)	4.2 (6.8)	0.415
	**30 s**	**Tpu_2.5 mm**	8.0 (4.2)	5.1 (6.8)	0.488
	**0 s**	**Tpu_1.0 mm**	8.3 (5.6)	6.1 (10.7)	0.735
	**10 s**	**Tpu_1.0 mm**	8.2 (5.5)	6.7 (9.5)	0.800
	**15 s**	**Tpu_1.0 mm**	7.9 (5.0)	7.1 (8.7)	0.878
	**30 s**	**Tpu_1.0 mm**	8.0 (4.2)	7.3 (8.1)	0.883

For the p-value computation different thicknesses of biomaterials have been considered. The SD is the standard deviation of temperature measurements.

Supplementary Table S2. Statistical analysis of temperatures means between control and PMS under the biomaterial samples in D_2

**Distance**	**Time**	**Biomaterial**	**Control mean (SD)**	**Biomaterial mean (SD)**	**pvalue**
**D_2**	**0 s**	**Med_2.5 mm**	21.9 (3.8)	23.7 (5.1)	0.594
	**10 s**	**Med_2.5 mm**	21.6 (3.4)	23.4 (5.1)	0.580
	**15 s**	**Med_2.5 mm**	21.6 (3.3)	23.1 (5.2)	0.628
	**30 s**	**Med_2.5 mm**	21.2 (3.2)	22.8 (5.3)	0.629
	**0 s**	**Med_1.0 mm**	21.9 (3.8)	23.1 (5.9)	0.743
	**10 s**	**Med_1.0 mm**	21.6 (3.4)	22.7 (6.0)	0.755
	**15 s**	**Med_1.0 mm**	21.6 (3.3)	22.6 (6.0)	0.780
	**30 s**	**Med_1.0 mm**	21.2 (3.2)	21.9 (5.9)	0.843
	**0 s**	**Tpu_2.5 mm**	21.9 (3.8)	23.8 (3.5)	0.495
	**10 s**	**Tpu_2.5 mm**	21.6 (3.4)	23.6 (3.5)	0.451

	**15 s**	**Tpu_2.5 mm**	21.6 (3.3)	23.4 (3.5)	0.477
	**30 s**	**Tpu_2.5 mm**	21.2 (3.2)	22.8 (3.5)	0.529
	**0 s**	**Tpu_1.0 mm**	21.9 (3.8)	26.0 (2.9)	0.137
	**10 s**	**Tpu_1.0 mm**	21.6 (3.4)	25.5 (3.2)	0.145
	**15_s**	**Tpu_1.0 mm**	21.6 (3.3)	25.4 (3.1)	0.142
	**30 s**	**Tpu_1.0 mm**	21.2 (3.2)	25.2 (2.9)	0.117

For the p-value computation different thicknesses of biomaterials have been considered. The SD is the standard deviation of temperature measurements.
